# N-Acetylcysteine for Cardiac Protection During Coronary Artery Reperfusion: A Systematic Review and Meta-Analysis of Randomized Controlled Trials

**DOI:** 10.3389/fcvm.2021.752939

**Published:** 2021-11-19

**Authors:** Sher Ali Khan, Ashley M. Campbell, Yingying Lu, Lingling An, Joseph S. Alpert, Qin M. Chen

**Affiliations:** ^1^Department of Pharmacy Practice and Science, College of Pharmacy, University of Arizona, Tucson, AZ, United States; ^2^Graduate Interdisciplinary Program in Statistics and Data Science, University of Arizona, Tucson, AZ, United States; ^3^Department of Biosystems Engineering, College of Agriculture and Life Sciences, University of Arizona, Tucson, AZ, United States; ^4^Department of Epidemiology and Biostatistics, Mel & Enid Zuckerman College of Public Health, University of Arizona, Tucson, AZ, United States; ^5^Department of Medicine and the Sarver Heart Center, University of Arizona College of Medicine, Tucson, AZ, United States

**Keywords:** N-acetylcysteine, coronary artery bypass, percutaneous coronary intervention, atrial fibrillation, antioxidants, reactive oxygen species, acute coronary syndrome, stable angina

## Abstract

Coronary artery reperfusion is essential for the management of symptoms in the patients with myocardial ischemia. However, the benefit of reperfusion often comes at an expense of paradoxical injury, which contributes to the adverse events, and sometimes heart failure. Reperfusion is known to increase the production of reactive oxygen species (ROS). We address whether N-acetylcysteine (NAC) reduces the ROS and alleviates reperfusion injury by improving the clinical outcomes. A literature search for the randomized controlled trials (RCTs) was carried out in the five biomedical databases for testing the effects of NAC in patients undergoing coronary artery reperfusion by percutaneous coronary intervention, thrombolysis, or coronary artery bypass graft. Of 787 publications reviewed, 28 RCTs were identified, with a summary of 2,174 patients. A meta-analysis using the random effects model indicated that NAC administration during or prior to the reperfusion procedures resulted in a trend toward a reduction in the level of serum cardiac troponin (cTn) [95% *CI*, standardized mean difference (SMD) −0.80 (−1.75; 0.15), *p* = 0.088, *n* = 262 for control, 277 for NAC group], and in the incidence of postoperative atrial fibrillation [95% *CI*, relative risk (RR) 0.57 (0.30; 1.06), *p* = 0.071, *n* = 484 for control, 490 for NAC group]. The left ventricular ejection fraction or the measures of length of stay in intensive care unit (ICU) or in hospital displayed a positive trend that was not statistically significant. Among the nine trials that measured ROS, seven showed a correlation between the reduction of lipid peroxidation and improved clinical outcomes. These lines of evidence support the potential benefit of NAC as an adjuvant therapy for cardiac protection against reperfusion injury.

## Introduction

A reperfusion injury has long been an unavoidable complication of the coronary artery revascularization procedures for the patients with acute or chronic myocardial ischemia. Although essential for the survival or for the relief of symptoms, reperfusion can contribute to as much as 40% of the final infarct size (1). The most common reperfusion procedure for the patients with myocardial ischemic is percutaneous coronary intervention (PCI). When reperfusion cannot be achieved successfully by PCI alone or in the setting of multivessel coronary disease, open heart surgery of coronary artery bypass graft (CABG) may be performed. Thrombolytic therapy can be prescribed during PCI, or alone when PCI and CABG are not readily available or impossible to perform due to the condition of a patient. One complication for each of these reperfusion treatments is periprocedural myocardial injury (PMI), which is linked to arrhythmias or reinfarction and in some cases heart failure. The release of massive amounts of reactive oxygen species (ROS) during reperfusion is thought to be an important cause of PMI.

Periprocedural myocardial injury is measurable with a number of clinical parameters, such as elevation of circulating cardiac troponins (cTn) or creatine kinase muscle band (CK-MB). Whereas, the amplitude or duration of cTn elevation can be predictive for the adverse events and heart failure (2–4), the extent of PMI is associated with the incidence of post-operative atrial fibrillation (POAF) (5). As a common complication following an open-heart surgery, the incidence of POAF can reach up to 70% in the patients after an elective CABG (6). POAF can cause stroke and increase the length of stay (LOS) in the intensive care unit (ICU) or in hospital. There is evidence supporting the concept that ROS and cytokine storm play a key role in the pathogenesis of POAF (7).

Despite a well-established association, ROS remains a neglected therapeutic target for the patients undergoing coronary reperfusion procedures. Administration of N-acetylcysteine (NAC) before reperfusion is expected to reduce the ROS generation. While a few randomized controlled trials (RCTs) showed a significant inhibition of cTn or CK-MB release or the incidence of POAF, other RCTs did not report positive outcomes. Given these inconsistences, it is prudent to address whether NAC provides a benefit for the coronary reperfusion procedures through a systematic review and meta-analysis approach.

A few meta-analyses have assessed the cardioprotective effect of NAC during cardiac surgery (8–12). However, each of these reports has a limited number of references. More importantly, none of these reports have included consideration of PCI. About 90% of the patients with ST segment elevation myocardial infarct (STEMI) and 50% of the patients with non-STEMI are treated with PCI (13), supporting the importance of PCI when considering the benefit of NAC during reperfusion. Nevertheless, none of these published meta-analyses have determined the impact of NAC on all the common clinical measures, such as elevation of cTn or CK-MB, change in left ventricular ejection fraction (LVEF), and ICU or hospital length of stay (LOS). In addition, whether the clinical outcomes correlate with the reduction of ROS has not been determined. Here, we address the cardioprotective effect of NAC when administered before PCI, CABG, or thrombolysis by summarizing the data from the publications with relevant clinical measures. In addition, the levels of antioxidants and ROS are captured to support the cause-effect relationship.

## Methods

The Preferred Reporting Items for Systematic Reviews (PRISMA) guideline was adopted for this systematic literature review using an *a-priori* inclusion and exclusion criteria (14).

### Inclusion and Exclusion Criteria

*A-priori* inclusion criteria were: (1) the RCTs assessing the effect of NAC in the patients >18 years old who underwent coronary reperfusion by PCI, CABG, or thrombolysis; (2) NAC was administered within 24 h before or during coronary reperfusion; (3) the RCTs should have measured the effect of NAC in comparison to a control group; (4) the control group should have received either placebo or standard care; (5) the published manuscripts and abstracts for the RCTs; (6) the RCTs published in any language; (7) the RCTs should not have selectively included the participants with any degree of renal insufficiency; and (8) the RCTs published from inception to September 18, 2021.

We excluded those RCTs in which the effect of NAC was not compared with a control group, but instead was compared with another pharmacologic agent. In addition, we excluded those RCTs reporting the trials designed for the selective patients with renal insufficiency, since renal insufficiency itself causes increased levels of cTn and CK-MB (15), potentially underestimating the beneficial effect of NAC on cardiac injury.

We considered both the clinical cardiac endpoints and mechanistic measures in this systematic review. The clinical endpoints included biomarkers of myocardial injury (cTn and CK-MB), cardiac contractility (left ventricular ejection fraction, LVEF), infarct size, incidence of POAF, and postoperative ICU or hospital LOS. The mechanistic measures consisted of markers for total antioxidant capacity (TAC) and ROS. To reduce the complexity of the data, we only extracted the serum and urine levels of the non-clinical markers and excluded the measures from the biopsy samples.

### Literature Search and Data Extraction

A comprehensive search strategy was developed with the assistance of a health science librarian (Rachel Walden) using a combination of keywords and controlled vocabulary to identify the studies reporting the use of NAC in the patients undergoing coronary artery reperfusion with PCI, CABG, or thrombolysis. The search strategy was developed for PubMed/Medline (NLM) and was subsequently translated to carry out the searches in four other biomedical bibliographic databases: Embase (Elsevier), Web of Science (Clarivate Analytics), Cumulative Index to Nursing and Allied Health Literature (CINAHL), and Cochrane Library (Wiley). In addition to searching the five bibliographic databases, a search of the gray literature (Clinicaltrials.gov) was performed. We searched for the trials from inception through September 18, 2021.

The following keywords were used to create the search strategy: myocardial reperfusion, T-Plasminogen activator, TPA, activase, alteplase, percutaneous transluminal coronary angioplasty, coronary balloon angioplasty, transluminal coronary balloon dilation, percutaneous coronary revascularization, percutaneous coronary intervention, PCI, coronary artery bypass grafting, CABG, aortocoronary bypass, coronary artery bypass surgery, coronary artery bypass, and acetylcysteine (as shown in Supplementary Material for full search strategy).

**Figure 1:**
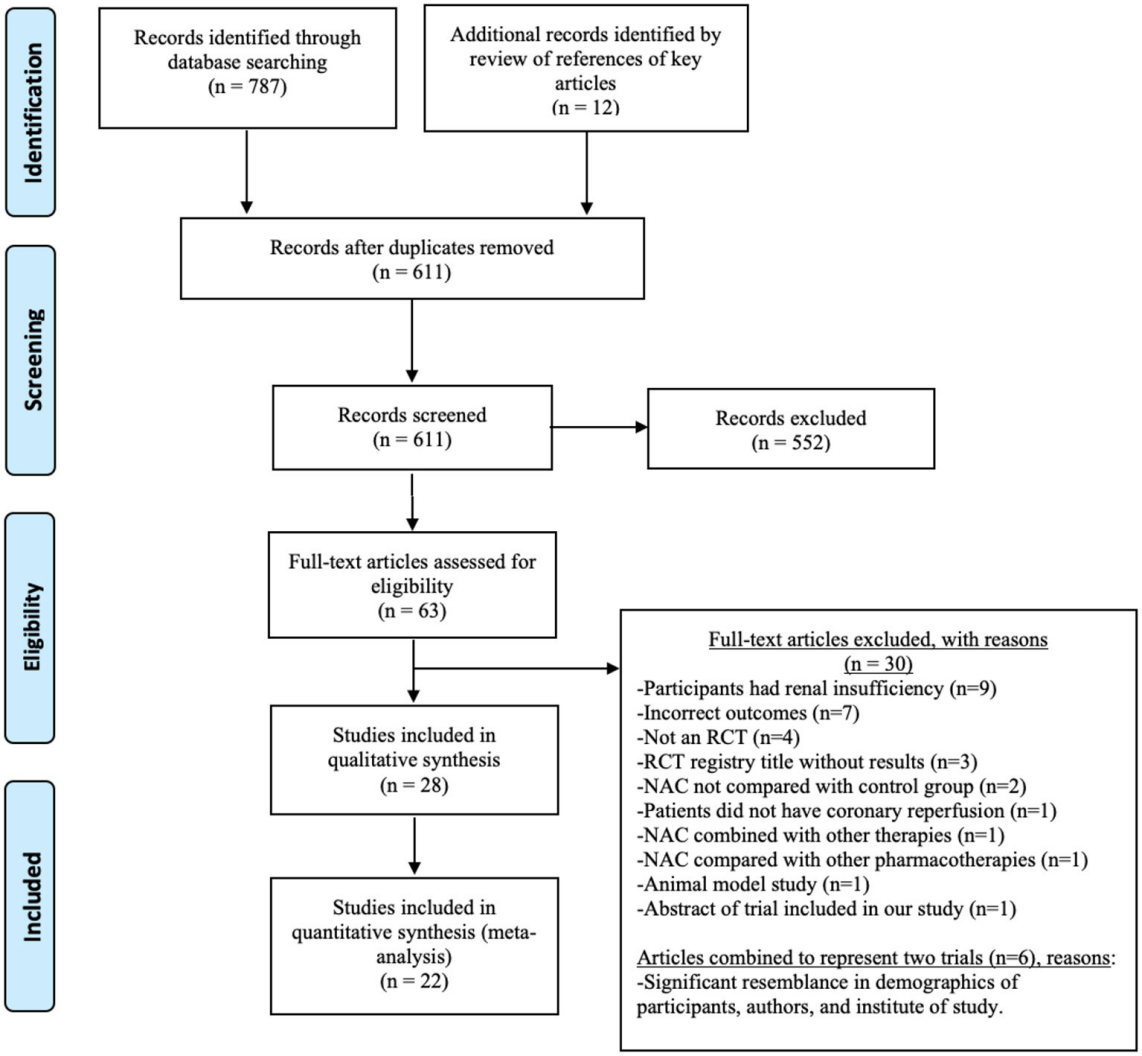
Preferred Reporting Items for Systematic Reviews and Meta-Analyses (PRISMA) flow diagram. The numbers document the literature search results.

The primary (SAK) and the secondary reviewer (AMC) independently searched and screened the reports. Rayyan QCRI Systematic Reviews Web Application was used after careful removal of duplicate records (16). No major discrepancies were noted among the two independent reviewers in the shortlisted trials. The primary reviewer extracted the data and assessed the risk of bias for each RCT, while the secondary reviewer validated the data for each publication. Minor discrepancies were noted in the extracted data, which were resolved with discussion reaching a mutual agreement. The PRISMA flowchart summary is shown in Figure 1.

### Quality Assessment of Included Trials

The revised Cochrane risk of bias tool for randomized trials (RoB2) was applied by the primary and secondary reviewers to assess the risk of bias for each included trial [https://methods.cochrane.org/bias/resources/rob-2-revised-cochrane-risk-bias-tool-randomized-trials (2020, accessed 10 May 2020)]. The following domains were evaluated: random sequence generation, allocation concealment, blinding of patients and personnel, blinding of outcome assessment, and incomplete outcome data. Similar to the data extraction process, minor discrepancies in the risk of bias assessment were resolved through discussion for consensus generation.

### Statistical Analysis

The measurements in cTn, CK-MB, LVEF, and LOS were treated as the continuous variables with reported means and SDs, while the incidence of POAF was treated as a dichotomous variable. Instead of applying the fixed effects models, which operate under the assumption that the estimated effects across the studies were pulled from a single population, we employed the random effects models to calculate the pooled effects, as the true effect may derive from a distribution, due to the fact that multiple studies were pulled from the different populations (17). The Sidik–Jonkman estimator was used for estimating the variance of the distribution of the true effect (18). The false positive rate increases when a small number of studies are enrolled and the outcome measures vary largely between the trials (19, 20).

In this NAC meta-analysis, the number of studies for each of six types of outcome measures varied from 5 to 12. Therefore, the Hartung-Knapp-Sidik-Jonkman method was also utilized to generate more robust estimates of the variance of pooled effects (19). When the outcome measures were continuous variables, the standardized mean difference (SMD) was calculated as a measure of effect size, as this is appropriate when different units were used across the studies (21). The SMD standardizes outcome measures in various units so that they are comparable at the same scale. Within a study, the SMD divides the mean difference of values of a measure by the pooled SD, thus SMD becomes a unitless standardized value. Hence, the SMDs can be compared across the studies for the related measures without the consideration of their respective units. The meta-analysis produces a pooled SMD, which denotes a change in the combined SD for a specific measure.

For dichotomous variables, relative risk (RR), a measure of effect size, was used as the likelihood of an event occurring between the two groups (NAC vs. control). The between-study heterogeneity was reported by I^2^. The individual effect size for each study and its weight, as well as confidence interval (CI) for the individual studies and pooled estimates, were included in the results. All the statistical analyses were performed using R version 3.6 (https://www.r-project.org/) (2013, accessed 10 May 2020). Specifically, the meta-analysis was performed by the packages meta (22), metafor (23), and dmetar (24).

## Results

### Characteristics of the Trials

Figure 1 shows the PRISMA flowchart and the number of publications evaluated, leading to the selection of 28 trials in 32 publications for this systematic review (25–56). The characteristics of the included trials are summarized in Table 1. Geographically, the reported trials were carried out in 10 countries: Turkey (10), Iran (5), India (3), Germany (2), Uzbekistan (1), Czech Republic (1), Finland (1), Canada (1), Australia (1), Brazil (1), Korea (1), and China (1). The total sample size, by adding the number of patients in the final statistical analyses for each of the 28 included trials, was 2,174. Among the 26 trials with the gender and age distribution indicated as shown in Table 1, the mean age of the patients ranged from 53 to 71.5 years old. The two trials did not disclose the age distribution (30, 56).

**Table 1:** The characteristics of 28 randomized controlled trials (RCTs) meeting the inclusion and exclusion criteria.

**References**	**Origin**	**Procedure**	**n (Ctr, NAC)**	**Age [Yr, Mean** **±** **SD, or** ***median (IQR)*****]**		**Sex (male)** ***n*** **(%)**		**Route**	**Ctr Tx**	**NAC dose**
				**Ctr**	**NAC**	**Ctr**	**NAC**			
Shafiei et al. (25)^a^	Iran	CABG^o^	58 (30, 28)	61.6 ± 7.7	57.7 ± 11.2	14 (46.7)	18 (64.3)	PO	PLB	4.2 g
Soleimani et al. (26)^c^	Iran	CABG^o^	141 (69, 72)	60.7 ± 8.4	62.4 ± 8.9	34 (49.2)	39 (54.1)	IV	PLB	0.05 g/kg
Pasupathy et al. (27)^d^	Australia	PCI^p^	112 (59, 53)	63 ± 14	64 ± 15	31 (52.5)	33 (62.2)	IV	PLB	1.2 g
Aldemir et al. (28)^e^	Turkey	CABG^o^	60 (30, 30)	*70.50 (68–73.2)*	*71.5 (69–73.5)*	22 (73)	18 (60)	IV	PLB	0.15 g/kg
Erdil, et al. (29)	Turkey	CABG^o^	82 (40, 42)	58.8 ± 9.9	58.6 ± 10.1	36 (85)	35 (83.3)	PO, IV	PLB	0.6 g/d ×3 d, 0.3 g
Nizomov et al. (30)^n^	Uzbekistan	PCI^p^	52 (25, 27)	NA	NA	NA	NA	NA	PLB	NA
Jalakandan et al. (31)	India	CABG^o^	50 (25, 25)	56.5 ± 6.7	59.8 ± 8.1	21 (84)	18 (72)	IV	PLB	0.15 g/kg
Talasaz et al. (32)^n^ Nozari et al. (33)^b^	Iran	PCI^p^	100 (50, 50)	58.3 ± 11.3	57.6 ± 11.5	36 (72)	42 (84)	IV IC	PLB	IV 0.1 g/kg/30 mins +IC 480 mg/20 mins+IV10 mg/kg/h for 12 h
Talasaz et al. (34)^b^	Iran	PCI^p^, TL^p^	88 (38, 50)	*61 (40–86)*	*61 (42–92)*	31 (82)	41 (82)	PO	PLB	1.2 g/d ×3 d
Kazemi et al. (35)	Iran	CABG^o^	240 (120, 120)	58.2 ± 12.7	61.3 ± 9.8	88 (73.3)	91 (75.8)	PO	PLB	1.2 g
Ozaydin et al. (36, 37)^f^	Turkey	CABG^o^	208 (104,104)	62 ± 9	63 ± 9	76 (73.1)	81 (77.9)	IV	PLB	0.05 g/kg
Kim et al. (38)	Korea	CABG^o^	48 (24, 24)	65.3 ± 7.6	60.8 ± 8.4	22 (91.6)	21 (87.5)	IV	PLB	0.1 g/kg
Buyukhatipoglu et al. (39)	Turkey	PCI^o^	60 (30, 30)	61.8 ± 10.0	58.9 ± 11.1	21 (70)	21 (70)	IV	Std	0.6 g
Kurian et al. (40)	India	CABG^o^	50 (25, 25)	60.1 ± 9.4	61.1 ± 10.3	17 (68)	15 (60)	IV	PLB	0.02 g/kg
Thiele et al. (41)^g^	Germany	PCI^p^	251 (125, 126)	*68 (57–75)*	*68 (56–76)*	82 (66)	89 (71)	IV	PLB	1.2 g
Prabhu et al. (42)	India	CABG^o^	53 (25, 28)	53.0 ± 8.1	54.2 ± 9.9	NA	NA	IV	Std	0.05 g/kg
Rodrigues et al. (43)^h^	Brazil	CABG^o^	20 (10, 10)	53 ± 7	54 ± 11	4 (40)	6 (60)	IV	Std	0.3 g
Köksal et al. (44)	Turkey	CABG^o^	30 (15, 15)	62.9 ± 4.9	63.4 ± 5.9	13 (86.6)	11 (73.3)	IV	Std	0.6 g
Ozaydin et al. (45) Peker et al. (46)	Turkey	CABG^o^	115 (57, 58)	59 ± 9	57 ± 11	44 (77.2)	47 (81)	IV	PLB	0.05 g/kg
El-Hamamsy et al. (47)	Canada	CABG^o^	100 (50, 50)	61.3 ± 7.4	59.8 ± 7.8	46 (92)	43 (86)	PO, IV	PLB	0.6 g, 0.05 g/kg
Koramaz et al. (48) Karahan et al. (49)	Turkey	CABG	44 (23, 21)	56.4 ± 3.1	58.6 ± 2.7	13 (56.5)	12 (57.1)	IV	Std	0.05 g/kg
Orhan et al. (50)	Turkey	CABG^o^	20 (10, 10)	61.8 ± 4.32	59.6 ± 5.48	6 (60)	7 (70)	IV	PLB	0.05 g/kg
Fischer et al. (51)^i^	Germany	CABG^o^, ^p^	40 (20, 20)	66.5 ± 6.5	66.2 ± 11.8	19 (95)	12 (60)	IV	PLB	0.1 g/kg
Sucu et al. (52)	Turkey	CABG^o^	40 (20, 20)	64 ± 6	66 ± 4	14 (70)	15 (75)	IV	PLB	0.050 g/kg/d ×3 d
Eren et al. (53)	Turkey	CABG^o^	20 (10, 10)	60.5 ± 5.7	61.1 ± 4.8	7 (70)	8 (80)	IV	PLB	0.1 g/kg
Vento et al. (54)^j^	Finland	CABG	35 (20, 15)	60.2 ± 1.7	63.1 ± 1.9	20(100)	15(100)	IV	Std	0.098 g/kg
Sochman et al. (55)^k^	Czech	TL^p^	30 (16, 14)	54.2 ± 7.2	52.2 ± 14.3	NA	NA	IV	PLB	0.1 g/kg
Yang et al. (56)	China	TL^p^	27 (7, 20)	NA	NA	NA	NA	IV	Std	1.2 g

*Ctr, control group; NAC, N-acetylcysteine group; n, enrollment number; Yr, year/s; IQR, interquartile range; CABG, coronary artery bypass grafting; PCI, percutaneous coronary intervention; TL, thrombolysis; NA, not available; IV, intravenous; IC, intracoronary; PO, per oral; PLB, placebo; Std, standard care; g, gram or grams; Kg, kilogram or kilograms; g/kg, gram of NAC per kg of body weight; d, day or days. The numbers without italic indicate mean +/− standard deviation (SD), whereas the numbers with italic indicate median with interquartile range (IQR) in parentheses*.

*The trial did not have a funding source unless indicated by ^“a−m^″*,

“a″ *funding from the Research Deputy of Bushehr University of Medical Science, Iran;*

“b″ *funding from the Tehran Heart Center, Tehran University of Medical Sciences;*

“c″ *the Research Deputy of Mazandaran University of Medical Sciences;*

“d″ *funded by the Australian National Heart Foundation*,

“e″ *funding from the University Scientific Research Projects Unit;*

“f″ *Daiichi-Sankyo Co provided test-kits for TAC and TOS levels;*

“g″ *funding from the University of Leipzig;*

“h″ *funded by Fundação de Amparo à Pesquisa do Estado de São Paulo and Fundação de Apoio ao Ensino, Pesquisa e Assistência do Hospital das Clínicas da Faculdade de Medicina de Ribeirão Preto-USP;*

“i″ *funding from the German Research Foundation;*

“j″ *funded by the Helsinki University Central Hospital; and*

“k″ *indicates funding by the Internal Grant Agency of the Ministry of Health of the Czech Republic*.

*All the trials are journals articles unless indicated by*

“n″ *which indicates abstract. Under the procedure for CABG, PCI, or TR*,

“o″ *indicates an elective procedure for stable atherosclerotic coronary artery disease*,

“p″ *indicates an emergency procedure for unstable atherosclerotic coronary artery disease*.

N-acetylcysteine was administered *via* intravenous (IV) infusion in the 23 trials and the oral route (PO) in the 3 trials. The two trials administered NAC *via* both IV and PO. One trial did not report the route of NAC administration or dose (30). The dose of NAC ranged from 20 to 150 mg/kg in the 19 trials, and 0.3–4.2 g in nine trials (Table 1). NAC was administered during coronary reperfusion in the 16 trials, while 8 trials administered NAC within 30–120 min before the start of reperfusion procedures. Four trials administered NAC the same day but before reperfusion procedure without specifying the timing (25, 28, 30, 34).

Among the 32 publications for the 28 trials included, 30 were journal articles and 2 were published abstracts. Twenty trials assessed the effect of NAC during CABG, five during PCI, two during thrombolysis, and one trail during PCI in combination with thrombolysis. Twenty-one trials had placebo controls, whereas seven practiced standard care in the control group. CABG was mostly elective for coronary artery disease, whereas the PCI and thrombolysis cases were urgent for acute coronary syndrome, except one trial where PCI was elective (39). All the included trials were published in English except one in Chinese (56), which was translated to English.

### Risk of Bias Analysis

**Figure 2:**
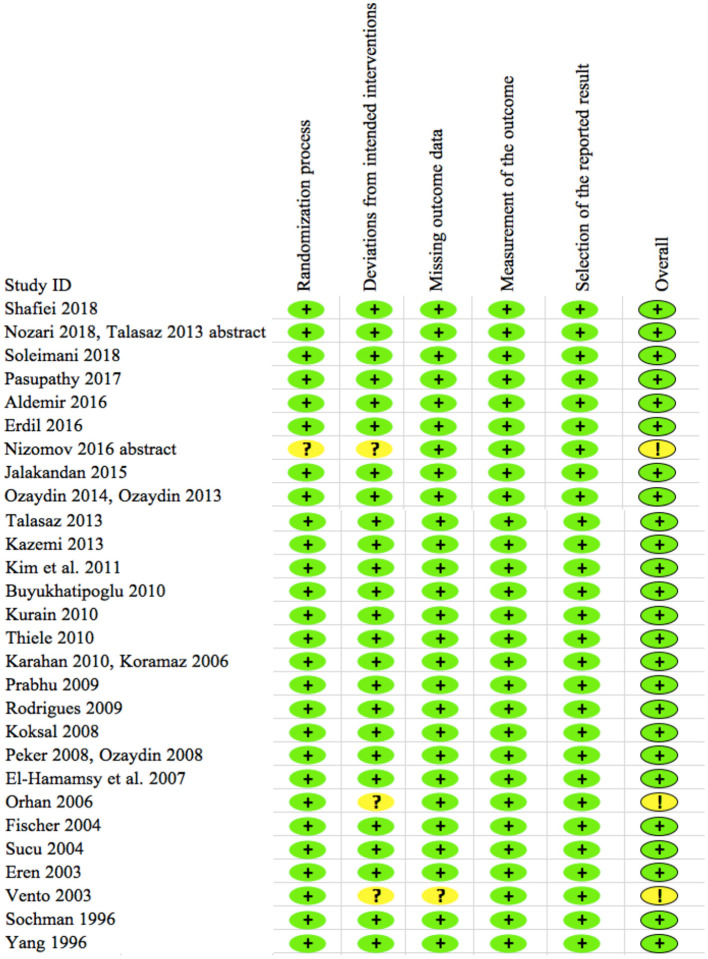
Risk of bias of 28 included trials. The plus sign in green (+) shows “low risk” for bias and the question or exclamation mark in yellow (?/!) shows “some concerns” for bias. None of the trials show high risk for bias per RoB2 analyses.

The results from the risk of bias analysis are indicated in Figure 2. Each domain was assigned with a low, unclear, or high risk of bias score. Among the 28 included trials, low risk of bias was noted in the 25 trials, while some concern for risk of bias was noted in 3 trials as indicated in Figure 2. None of the trials showed a high risk of bias. Hence all the trials were included for the synthesis of final results.

### Effect of NAC Administration on the Clinical Outcomes

#### Serum cTn Elevation

**Figure 3:**
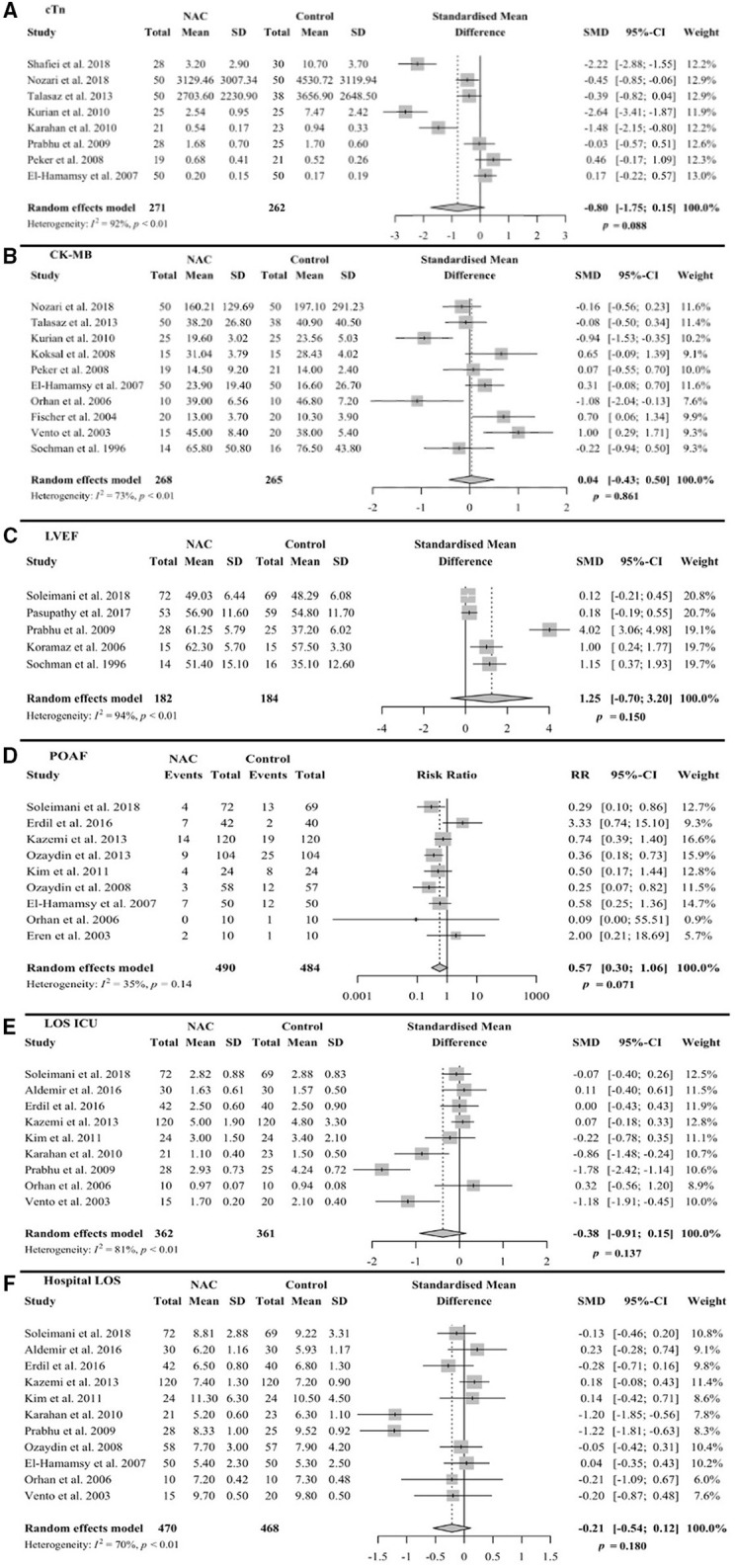
Forest plots of random effects model meta-analysis with 95% confidence interval (CI) comparing NAcetylcysteine (NAC) group vs. control group. The plots are showing standard mean difference (SMD) for continuous variables along with standard deviations (SD) or risk ratio (RR) for binary variables along with events for **(A)** serum troponin levels at 24 h after procedure. Shafiei et al. (25) measured cTnI in ng/ml, Nozari et al. (33) measured high sensitivity TnT (hs-TnT) in ng/dl, Talasaz et al. (32, 34) measured hs-TnT (unit not available, inquires not answered), Kurian et al. (40) measured cTnI in ng/ml, Karahan et al. (49) measured cTnT in ng/ml, Prabhu et al. (42) measured cTnI in ng/ml, Peker et al. (46) measured cTnT in ng/ml, and El-Hamamsy et al. (47) measured cTnT in ng/l (per response to inquires). **(B)** Serum CK-MB levels, **(C)** left ventricular ejection fraction (LVEF), **(D)** post-operative atrial fibrillation (POAF), **(E)** length of stay (LOS) in intensive care unit (ICU), and **(F)** LOS in hospital.

Eight trials reported the means and SDs for the serum levels of either cardiac troponin I (cTnI) or troponin T (cTnT) following CABG or PCI (25, 33, 34, 40, 42, 46, 47, 49). The units of the measures are indicated in Figure 3 legend. Two of the reports did not include units for troponin (34, 47). The inquires to the authers of one report were not answered. A meta-analysis using SMD allows us to pool the values of cTnI and cTnT in a scaleless format into one analysis (57). This method does not require units for troponin. The means and SDs were extracted from each trial for the meta-analysis, with the form of troponin measured from each trial indicated in the figure legend (Figure 3A). Adding the enrollments from these trials yielded a total number of 271 for NAC and 262 for the control group. With a 95% *CI*, the pooled SMD was −0.80, with a range from −1.75 to 0.15 (*p* = 0.088). The value −0.8 implies that cTn decreased by 0.8 times the pooled SD, which was 1.1, as a result of the NAC treatment when compared with placebo or standard care. This indicates a notable decrease in the cTn levels, even though the *p*-value for such decrease is 0.088, not significant but showing a trend. As expected, a high heterogeneity was observed across the trials (I^2^ = 92%, *p* < 0.01).

One trial was not entered into the meta-analysis due to reported median and interquartile ranges (IQR) for cTn, instead of means and SDs (43); hence, ineligible for grouping with the rest of the studies to perform the meta-analysis. This study used low dose NAC, 0.3 g, and did not indicate whether the reduction in the median cTn levels was significant due to NAC treatment [NAC group 4.8 (IQR 2.7, 6.0)] vs. control [5.5 (IQR 2.8, 6.4)]. Overall, our meta-analysis of eight trials suggests that there is a trend toward the reduced levels of cTn in the NAC group when compared with the control group with 95% *CI*.

#### Serum CK-MB Elevation

The means and SDs for the serum CK-MB concentrations were reported in 10 trials following CABG, PCI, or only pharmacological therapy (33, 34, 40, 44, 46, 47, 50, 51, 54, 55). With a meta-analysis using a 95% *CI*, we obtained a SMD value of 0.04, ranging from −0.43 to 0.50 (*p* = 0.861) (Figure 3B). The heterogeneity was moderate across the trials (I^2^ = 73%, *p* < 0.01). One trial was not compatible for the meta-analysis, due to reporting median and IQR instead of the means and SDs. This trial indicated no significant difference between the NAC and control groups [338 (IQR 290, 383) vs. 313 (IQR 260, 356) μmol/L/h, *p* = 0.13] (41). Overall, the NAC treatment had no significant effect on the procedure-associated elevation of CK-MB in the serum.

#### Infarct Size

Three trials measured the infarct size after a coronary reperfusion procedure at 7 days (27, 41, 55). The infarct size was measured using cardiac magnetic resonance imaging (CMR) (27, 41) or electrocardiography (55). While one trial showed no significant difference [NAC group 17.4% (IQR 9.1, 25.9, *n* = 126) vs. control group 14.3% (IQR 8.0, 26.2, *n* = 125), *p* = 0.47] (41), the two trials reported significantly smaller infarct size in the NAC vs. control groups [11% (IQR 4.1, 16.3, *n* = 53) vs. 16.5% (IQR 10.7, 24.2, *n* = 59), *p* = 0.02; *or* 16.3 ± 10.5, *n* = 14 vs. 24.4% ± 9.5, *n* = 16, *p* < 0.05) (27, 55). Furthermore, Pasupathy et al. (27) measured infarct size at 3 months and reported a significant reduction with the NAC treatment, with the infarct size in the NAC group being 5% on average (IQR 0.7, 12.4, *n* = 26) compared with the control group, which was 10.2% (IQR 6.8, 14.8, *n* = 29 *p* = 0.02). Overall, the two trials showed significant reduction of infarct size, while one trial showed no significant reduction; hence, the results are inconclusive with regard to whether or not NAC can reduce the infarct size.

#### Left Ventricular Ejection Fraction

Left ventricular ejection fraction was measured within 7 days after coronary reperfusion in the five trials with a sum of 182 enrollments for NAC and 184 for the control group (26, 27, 42, 48, 55). The reported means and SDs were used for the meta-analysis (Figure 3C). With a 95% *CI*, the SMD was 1.25 with a range of −0.70 to 3.20 (*p* = 0.150). The heterogeneity was high across the trials (I^2^ = 94%, *p* < 0.01). Although the statistical results do not support that NAC had a significant influence on the LVEF, the distribution of means plus SMD value point to a trend of NAC benefit in improving the LVEF.

Not included in the meta-analysis were 4 trials, with a total enrollment of 442, due to reported median instead of means or the differences in time points of the LVEF measurements (27, 30, 41, 56). Nizomov et al. (30) measured LVEF at 1- and 3-month after PCI and indicated a significantly smaller number of participants with LVEF <50% in the NAC vs. control groups [11% vs. 16% at 1 month, *p* = 0.046, and 4% vs. 16% at 3 months, *p* = 0.017], suggesting a benefit of the NAC treatment. Thiele et al. (41) reported that the median values of LVEF measured at 7 days were not significantly different [NAC 52.1% (IQR 43.5, 59.2) vs. control 50.6% (IQR 41.6, 58.6), *p* = 0.23]. Pasupathy et al. (27) did not find a significant difference (NAC 59.6 ± 11.1% vs. control 56.7 ± 10.5%, *p* = 0.33) in LVEF measured at 3 months. Yang et al. (56) neither revealed the time point of measurement nor reported significant difference in LVEF between the NAC and control groups (57 vs. 53%, no SDs or *p*-values provided). Overall, the results are inconclusive based on the reported median values of LVEF.

#### Post-operative Atrial Fibrillation

The incidence of POAF was reported in the 9 trials after CABG with a total combined patient number of 490 for NAC and 484 for control (26, 29, 35, 36, 38, 45, 47, 50, 53). It is known that POAF is a rare event following PCI, providing an explanation for the lack of POAF in the PCI trials. The number of patients developing POAF after reperfusion was registered either during the postoperative ICU stay or during the first 3 days of hospital stay. Using the binary outcome of the meta-analysis due to the report of events, we obtained the relative risk (RR) value of 0.57 with 95% *CI*, ranging from 0.30 to 1.06 (*p* = 0.071). The heterogeneity was low across the trials (I^2^ = 35%, *P* = 0.14). The meta-analysis points to a reduction, close to 50%, in the incidence of POAF with NAC treatment (Figure 3D).

#### LOS in ICU

The nine trials reported the LOS in ICU with means and SDs for an added-up enrollment of 362 for the NAC group or 361 for the control group (26, 28, 29, 35, 38, 42, 49, 50, 54). The meta-analysis yielded SMD −0.38 with 95% *CI*, ranging −0.91 to 0.15 (*p* = 0.137, Figure 3E). The heterogeneity was high across the trials (I^2^ = 81%, *p* < 0.01). Although the meta-analysis results did not reveal a significant difference per 95% *CI*, there is a trend toward the reduction of LOS in ICU by the NAC treatment.

#### LOS in Hospital

Hospital LOS with means and SDs were reported in 11 trials with a total enrollment adding up to 470 (NAC treated) or 468 (control) (26, 28, 29, 35, 38, 42, 45, 47, 49, 50, 54). The meta-analysis produced a SMD of −0.21 with 95% *CI*, ranging −0.54 to 0.12 (*p* = 0.180), and high heterogeneity (I^2^ = 70%, *p* < 0.01) (Figure 3F). Similar to LOS in ICU, a trend toward the reduction in hospital LOS in the NAC group is shown by the upper boundary of the 95% *CI* close to 0.

### Effect of NAC on the Antioxidant Reservoir and ROS

Eighteen publications contained the measures for antioxidants and ROS, among which the nine trials had clinical outcome measures along with the lipid peroxidation product malondialdehyde (MDA). There is a lack of uniformity in the assays or time point of measurements between the studies, and most of the measures at a specific time point have less than five trials, which is not ideal for a meta-analysis. Nevertheless, for most of the measures, there was consistent reduction between the trials.

#### Total Antioxidant Capacity (TAC)

Seven trials measured the antioxidant levels after the coronary reperfusion procedures (31, 37, 39, 40, 42, 44, 56) (Table 2). These studies reported the levels of antioxidants at the baseline and different time points after coronary reperfusion, from 10 min to 48 h. The measurements included reduced glutathione (GSH) or the activities of glutathione peroxidase, glutathione reductase, superoxide dismutase, and catalase in the serum. Two trials reported the outcomes as total antioxidant capacity (TAC) without specifying the scaled measures (37, 39). One trial measured the urine levels of TAC in addition to the serum levels (39).

**Table 2:** Total antioxidant capacity (TAC) after coronary artery reperfusion.

**References**	**Measure**		**Baseline**	**10 min**	**30 min**	**1–12 h**	**24–48 h**
Jalakandan et al. (31)	GSH (nmol/ml)	Ctr	32.79 ± 15.78			24.25 ± 11.56	
		NAC	28.18 ± 10.14			33.82 ± 11.70	
		P	0.225			0.005	
Ozaydin et al. (37)	TAC^a^ (mmol Trolox/L)	Ctr	*1.6 (0.7–3.0)*				*1.4 (0.6–3.2)*
		NAC	*1.6 (0.7–2.9)*				*1.9 (0.9–3.9)*
		P	0.89				<0.0001
Buyukhatipoglu et al. (39)	serum TAC (mmol Trolox/L)	Ctr	0.84 ±0.14				0.77 ±0.09
		NAC	0.88 ±0.12				0.81 ±0.07
		P	NS				NS
	urine TAC (mmol Trolox/L)	Ctr	1.52 ± 0.10				1.47 ± 0.16
		NAC	1.56 ± 0.12				1.49 ± 0.10
		P	NS				NS
Kurian et al. (40)	glutathione peroxidase (U/g Hb)	Ctr	6.30 ± 1.2	6.33 ± 1.1	5.89 ± 0.9	5.26 ± 0.9	
		NAC	6.41 ± 1.1	5.41 ± 1.0	4.36 ± 0.8	5.26 ± 0.9	
		P	NS	NS	<0.05	NS	
	glutathione reductase (U/g Hb)	Ctr	1.08 ± 0.16	0.41 ± 0.07	0.42 ± 0.08	0.68 ± 0.08	
		NAC	1.106 ± 0.16	0.426 ± 0.07	0.496 ± 0.08	0.747 ± 0.08	
		P	NS	NS	<0.05	<0.05	
	Superoxide dismutase (U/g Hb)	Ctr	3829.1 ± 323	1218.6 ± 255	1258.9 ± 213	1375.9 ± 221	
		NAC	3938.8 ± 340	1264.7 ± 241	1334.1 ± 254	1461.8 ± 222	
		P	NS	NS	<0.05	<0.05	
	Catalase (pM H_2_0_2_ /min /g Hb)	Ctr	625.72 ± 20.5	985.27 ± 37.6	901.02 ± 36.1	869.93 ± 33.7	
		NAC	620.44 ± 21.73	955.87 ± 39.14	859.47± 35.22	741.38 ± 34.23	
		P	NS	NS	<0.05	<0.05	
Prabhu et al. (42)	GSH ^a^ (mg/g Hb)	Ctr	0.7 ± 0.08	1.3 ± 0.20		1.25 ± 0.18	1.21 ± 0.15
		NAC	0.75 ± 0.03	1.6 ± 0.10		1.66 ± 0.05	1.31 ± 0.14
		P	NS	<0.001		<0.01	NS
	Glutathione peroxidase^a^ (U/g Hb)	Ctr	42.6 ± 2.7	80.4 ± 6.4		59 ± 8	51.6 ± 5.6
		NAC	40.6 ± 3.4	85.7 ± 3.7		62.7 ± 2.7	55 ± 1.4
		P	NS	<0.01		NS	<0.05
	Glutathione reductase^a^ (μg/min/g Hb)	Ctr	8.6 ± 0.4	9.9 ± 0.48		10.1 ± 0.5	9.8 ± 0.4
		NAC	8.6 ± 0.4	10.5 ± 0.5		10.4 ± 0.4	9.5 ± 0.1
		P	NS	<0.001		<0.001	NS
	Superoxide dismutase ^a^ (U/g Hb)	Ctr	367 ± 33	644 ± 31		564 ± 31.8	531 ± 31
		NAC	377 ± 27	708 ± 15		582 ± 18	537 ± 32
		P	NS	<0.001		NS	NS
	Catalase ^a^ (μmol H_2_O_2_/ min/g Hb)	Ctr	3.7 ± 1.30	6.0 ± 0.42		5.8 ± 0.60	5.7 ± 0.30
		NAC	4.0 ± 1.0	6.4 ± 0.47		5.8 ± 0.10	5.4 ± 0.40
		P	NS	<0.01		NS	NS
Köksal et al. (44)	Glutathione peroxidase ^a^ (U/g Hb)	Ctr	24.3 ± 10.7		22.5 ± 8.9		
		NAC	27.7 ± 8.3		28.7 ± 12.9		
		P	NS		NS		
Yang et al. (56)	GSH (mol/L)	Ctr	2.0 ± 2.4			1.4 ± 0.3	1.4 ± 0.4
		NAC	2.2 ± 2.4			2.8 ± 1.3	2.8 ± 1.2
		P	NS			<0.05	<0.05

*All numbers represent means ± SDs unless they are italicized, which indicate median (with interquartile ranges, IQR). Ctr: control group, NAC: N-acetylcysteine group. NS: non-significant*.

“a″ *indicates that the value was extracted from the figures of the cited publication. TAC: total antioxidant capacity, GSH: reduced glutathione, U/g: units per gram, Hb: Hemoglobin, mmol: millimole(s), nmol: nanomole(s)*.

The measurements of GSH between 1 and 12 h showed significant increases in the three trials (Table 2). No significant differences in the activity of glutathione peroxidase were observed, but there was a slight improvement in glutathione reductase in the 2 trials (Table 2). The data on superoxide dismutase and catalase are inconsistent among the 3 trials (Table 2). Ozaydin et al. (37) reported an improvement of TAC at 24–48 h after NAC, but not by Buyukhatipoglu et al. (39). The latter trial used a much lower dose of NAC (0.6 g, which translates to 0.01 g/kg per 60 kg body weight) than the average dose of 0.1 g/kg dose. Overall, there is evidence to support the possibility that the administration of NAC before a coronary reperfusion procedure leads to an increase of glutathione redox system activity as expected.

#### Reactive Oxygen Species

Fourteen publications reported the levels of ROS markers after the coronary reperfusion procedures (25, 27, 31, 37, 39–42, 44, 48, 49, 52, 53, 56) (Table 3). The time points of measurements include the baseline and 15 min to 72 h after coronary reperfusion. The ROS was measured as MDA, myeloperoxidase (MPO) activity, oxidized glutathione (GSSG), advanced oxidation protein products (AOPP), or oxidized low density lipoprotein (LDL). The two trials reported the outcomes as the total oxidative stress (TOS) or total oxidant capacity (37, 39). All of these trials measured the serum levels of ROS markers except one trial, which also reported the urinary levels in addition to the serum levels (39).

**Table 3:** Total oxidative stress (TOS) after coronary reperfusion.

**References**	**Measure**		**Baseline**	**15–30 min**	**1–3 h**	**3–8 h**	**12 h**	**24 h**	**48–72 h**
Shafiei et al. (25)	MDA (nmol/ml)	ctr	35.96 ± 10.37	42.53 ± 12.37		45.13 ± 12.52			
		NAC	22.92 ± 4.33	14.11 ± 8.02		11.74 ± 6.17			
		p	NS	<0.05		<0.05			
Pasupathy et al. (27)	(log) MDA (μM)	ctr			0.81 ± 0.03				
		NAC			0.82 ± 0.03				
		p			<0.01				
	(log) MPO ^a^ (ng/ml)	ctr			2.31 ± 0.09				
		NAC			2.37 ± 0.06				
		p			0.64				
Jalakandan et al. (31)	MDA ^a^ (nmol/ml)	ctr	1.40 ± 0.63		2.26 ± 1.03				
		NAC	1.70 ± 0.87		1.58 ± 1.12				
		p	0.164		0.033				
Ozaydin et al. (37)	TOS ^a^ (mmol h_2_o_2_/L)	ctr	*19.2 (4.9–38.8)*						*24.2 (2.2–41.9)*
		NAC	*18.7 (3.0–65.0)*						*19.3 (4.0–41.0)*
		p	0.81						<0.0001
Buyukhatipoglu et al. (39)	Serum TOC (μmol H_2_O_2_/L)	ctr	13.80 ±3.64					20.38 ±5.58	
		NAC	15.35 ±4.30					18.90 ±5.58	
		p	NS					NS	
	Urine TOC (μmol H_2_O_2_/L)	ctr	19.46 ±5.96					28.99 ±9.23	
		NAC	21.02 ±7.17					29.27 ±7.99	
		p	NS					NS	
Kurian et al. (40)	MDA (nM/g Hb)	ctr	0.9 ± 0.11	3.379 ± 0.18	3.121 ± 0.18	2.324 ± 0.14			
		NAC	0.955 ± 0.10	2.685 ± 0.19	2.198 ± 0.11	1.501 ± 0.12			
		p	NS	NS	<0.05	<0.05			
Thiele et al. (41)	AOPP ^a^ (μmol/L) (fold of baseline)	ctr	*40.4 (27.5–54.3)*	1.025 ± 0.32				1.083 ± 1.12	0.9 ± 0.45
		NAC	*40.9 (29.9–58.9)*	0.9 ± 0.67				0.77 (NA)	0.85 (NA)
		p	0.3	NS				<0.05	NS
	oxidized LDL ^a^ (ng/ml) (fold of baseline)	ctr	*32.3 (12.7–141.8)*	1.07 ± 0.22				1.07 ± 0.34	1.12 ± 0.34
		NAC	*34.8 (16.4–95.1)*	0.91 ± 0.45				0.8 ± 0.45	0.83 ± 0.56
		p	0.94	NS				<0.05	<0.05
Karahan et al. (49)	MDA (nmol/ml)	ctr	1.46 ± 0.23			3.11 ± 0.70	2.81 ± 0.61	2.41 ± 0.56	2.04 ± 0.41
		NAC	1.45 ± 0.24			2.2 ± 0.38	1.85 ± 0.31	1.58 ± 0.27	1.46 ± 0.24
		p	0.909			<0.001	<0.001	<0.001	<0.001
Prabhu et al. (42)	MDA (nM/gHb)	ctr	15 ± 1.3	19 ± 2.5			17.5 ± 1.5	16 ± 1.3	
		NAC	14 ± 2.6	18 ± 2.3			16.5 ± 1.4	14 ± 1.2	
		p	NS	<0.05			<0.05	<0.001	
Köksal et al. (44)	MDA ^a^ (nmol/ml)	ctr	0.72 ± 0.13		0.89 ± 0.20				
		NAC	0.67 ± 0.13		0.76 ± 0.14				
		p	NS		<0.05				
Koramaz et al. (48)	MDA ^a^(nmol/ml)	ctr	1.62 ± 0.31			2.6 ± 0.15	2.6 ± 0.77	2.25 ± 0.50	2 ± 0.04
		NAC	1.5 ± 0.31			1.4 ± 0.12	1.4 ± 0.39	1.3 ± 0.31	1.1 ± 0.03
		p	NS			<0.05	<0.05	<0.05	<0.05
Sucu et al. (52)	MPO ^a^ U (mg protein)^−1^h^−1^	ctr	0.034 ± 0.01		0.062 ± 0.02	0.055 ± 0.02		0.038 ± 0.01	
		NAC	0.032 ± 0.01		0.04 ± 0.06	0.038 ± 0.01		0.031 ± 0.01	
		p	0.592		0.000	0.000		0.000	
	MDA ^a^ (nmol/ml)	ctr	7.1 ± 5.4		12.6 ± 5.7	14.75 ± 5.9		10.1 ± 4.7	
		NAC	7.5 ± 3.3		8.75 ± 2.9	10.25 ± 2.5		7.8 ± 2.8	
		p	0.675		0.000	0.000		0.000	
Eren et al. (53)	MDA (nmol/ml)	ctr	2.34 ± 0.31	2.84 ± 0.72					
		NAC	2.19 ± 0.42	2.51 ± 0.65					
		p	NS	0.043					
Yang et al. (56)	GSSH (mol/L)	ctr	0.15 ± 0.23			0.12 ± 0.08		0.11 ± 0.07	
		NAC	0.14 ± 0.11			0.08 ± 0.05		0.05 ± 0.03	
		p	NS			NS		<0.05	

*All numbers represent means ± SDs unless they are italicized, which indicate median (with IQRs). Ctr, control group; NAC, N-acetylcysteine group. NS, non-significant*.

“a″ *indicates that the values were extracted from figures of the cited reference. MDA, malanodealdehyde; TOC, total oxidant capacity; AOPPs, advanced oxidation protein products; MPO, myeloperoxidase; TOS, total oxidative stress; LDL, low density lipoprotein; GSSH, oxidized glutathione; Hb, Hemoglobin; g, gram(s); L, liter(s); ml, milliliter(s); nmol, nanomole(s)*.

Malondialdehyde was measured in the 9 trials, all of them showed significant reduction with the NAC treatment at different time points regardless of the reperfusion procedure performed, either PCI, CABG, or thrombolytic therapy (Table 3). MPO showed significant reduction in one trial but not the other (Table 3). Decreases of oxidized glutathione were observed in one trial (Table 3). Overall, there is evidence that the administration of NAC before the coronary reperfusion procedure significantly lowers the levels of ROS markers in the patients receiving NAC at various time points as compared with the control group.

### Correlation of ROS Reduction With the Clinical Outcomes

Table 4 compares NAC induced improvements in the TAC or ROS reduction with the clinical outcome measures. It is evident that a significant improvement of TAC or ROS reduction due to NAC correlates with the reduced levels of cTn, increased LVEF, and decreased LOS in ICU or hospital. Such correlation supports the cause-effect relationship of TAC or ROS with the improved clinical outcomes. This suggests that NAC might have mediated the improved clinical outcomes through the reduction of ROS.

**Table 4:** Correlation of reactive oxygen species (ROS) and TAC with the clinical outcomes.

**References**	***n***	**TAC**	**ROS**	**cTn**	**CK-MB**	**LVEF**	**POAF**	**LOS ICU**	**LOS hospital**
Shafiei et al. (25)	58		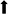 MDA	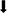					
Pasupathy et al. (27)	112		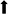 MDA			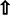			
Ozaydin et al. (36, 37)	172	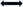 TAC	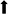 TOS				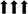		
Kurian et al. (40)	50	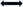 SOD, GR	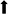 MDA		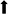				
Karahan et al. (49)	44		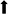 MDA					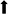	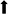
Prabhu et al. (42)	53	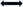 GSH	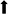 MDA	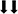		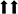	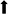	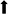	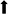
Köksal et al. (44)	30	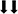 GPX	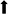 MDA		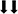				
Koramaz et al. (48)	30		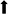 MDA	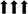		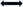		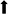	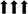
Eren et al. (53)	20		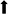 MDA				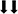		

*n, indicates sample size; ROS, reactive oxygen species; TAC, total antioxidant capacity; cTn, cardiac troponin; CK-MB, creatine kinase muscle band; LVEF, left ventricular ejection fraction; POAF, post-operative atrial fibrillation; LOS, length of stay; ICU, intensive care unit; SOD, superoxide dismutase; GR, glutathione reductase; GSH, reduced glutathione; GPX, glutathione peroxidase; MDA, malondialdehyde; TOS, total oxidative stress. 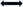
indicates increase. 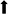 indicates decrease. 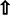
indicates minor increase. 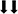
indicates no change*.

### Sensitivity Analysis

We performed a sensitivity analysis to assess both the between-study heterogeneity and publication bias to ensure that the pooled effects for meta-analysis were indeed robust (58, 59). Between-study heterogeneity may be caused by a trial with either an extreme enrollment size or a larger impact on the pooled effect. To detect an influential trial, the Cook's distance, a well-established influential point detection method, was used (60). A trial may be considered as an influential case if the Cook's distance is >0.45 (17). Supplementary Figure 1 shows the Cook's distance for each measure in the meta-analyses, with the potential influential study highlighted in red. For cTn, CK-MB, or POAF, none of the trials have a Cook's distance over 0.45, indicating that there is no influential trial. For LVEF, LOS ICU, or hospital LOS, one potential influential study was detected, which is by Prabhu et al. (42). To verify if the influential trial affects the summary data or the conclusion, we compared the results from the random-effects model with vs. without the influential trial. Removal of Prabhu et al. (42) trial reduced the heterogeneity for LVEF, LOS ICU, or hospital LOS, but did not improve the *p*-value or the direction of SMD (Supplementary Table 1), and therefore did not affect our conclusions.

Another potential issue for the meta-analysis is the publication bias due to the trials with a small sample size (17). We checked the small-study effects using the funnel plots, which display the relationship between the SMD of studies against its standard error (61). When there is no publication bias, the distribution of the trials in points (one point represents each trial) is symmetric and fits into the shape of an upside-down funnel. In the case of this NAC meta-analysis, a few trials landed outside the funnel area, but the asymmetry is not across all the different outcome measures (as shown in Supplementary Figure 2). Since visual inspection can be subjective, we performed the Egger's regression test (62) to evaluate the asymmetry quantitatively in the funnel plot for the continuous outcome measures, cTn, CK-MB, LVEF, LOS in ICU, and LOS in hospital, and Peters' regression test (63) for the binary outcome measure POAF. The results are shown in Supplementary Table 2. None of the statistical tests have a significance at the threshold of 0.05, suggesting that the funnel plots are roughly symmetrical. This indicates that the publication bias is not a major concern in the meta-analysis.

## Discussion

The administration of NAC prior to the coronary reperfusion procedures was associated with a trend toward the inhibition of cTn elevation, reduced incidence of POAF, and lowered levels of ROS. The decrease of cTn by NAC treatment is considered notable due to the summary SMD being −0.8 in reference to the SD of 1.1 from the meta-analysis of eight trials (Figure 3A). However, the overall *p*-value of 0.088 suggests that the decrease is close to 0.05 but not truly significant in the statistical analysis using 95% *CI*. While improvement in LVEF or reduction in ICU and hospital LOS were not statistically significant at 95% *CI*, the meta-analyses suggested a minor trend toward the improvement for these measures (Figures 3C,E,F). The effect of NAC on infarct size remains inconclusive due to the smaller number of trials. CK-MB represents the only outcome that did not show improvement with the administration of NAC. Given the fact that POAF is associated with older age and an increase in all-cause mortality (64), and whereas the level of cTn elevation predicts the incidence of adverse events and the risk of heart failure (2–4), adding NAC as an adjuvant therapy for reperfusion may provide benefit in these parameters. By decreasing these clinical complications, it could be expected that NAC administration might reduce the adverse events and the development of heart failure as well as possibly improving the long-term mortality.

An acute kidney injury (AKI) is often an additional complication of reperfusion procedures. We did not include this measure in our study due to the lack of such information in majority of the clinical trials on NAC for cardiac protection and the recently published systematic reviews with meta-analysis on the topic. Guo et al. (65) used the random effects model to evaluate the seven clinical trials for the effects of NAC on contrast-induced AKI in the patients with STEMI following PCI. This report showed a significantly reduced rate of AKI and all-cause hospital mortality with NAC compared with the placebo group (65). However, a meta-analysis of eight trials by Mei et al. using the random effects model for perioperative NAC among the patients with cardiac surgery concluded that there was no significant benefit in the prevention of AKI. The American College of Cardiology Foundation (ACCF) and American Heart Association (AHA) Guideline for Coronary Artery Bypass Graft Surgery noted the controversy surrounding the use of NAC for the prevention of CABG-associated AKI (66). However, the benefit of NAC as a potential intervention for POAF was not addressed.

Our data on POAF reduction with NAC are consistent with the published meta-analyses reporting the benefit of NAC for the patients with cardiac surgery. Two meta-analyses used the fixed effects model to determine the impact of NAC on POAF when administered before CABG among the eight trials, and showed a significant reduction of POAF (10, 12). In addition, the reduction of POAF was reported by Liu et al. (9), who summarized 10 publications (without the consideration of redundancy in trials) with meta-analysis using the fixed effects model. Wang et al. (11) registered seven trials for meta-analysis using the random effects model and discovered a trend toward improvement in the incidence of POAF with NAC.

The additional clinical measures are less convincing for the benefit of NAC examining in our data and that of others. Pereira et al. (8), compiled 12 trials for meta-analysis with the random effects model and showed a trend but not statistical significance toward an improvement in the post-operative cardiac insufficiency, ICU LOS, or hospital LOS, and incidence of post-operative acute myocardial infarction or cardiac arrhythmias. Gu et al. (10) did not find that NAC reduced ICU LOS using a fixed effects model for a meta-analysis of four trials. Similarly, Liu et al. (9) did not find significant improvement or a trend toward the improvement of ICU or hospital LOS with five trials. Wang et al. (11) showed neither statistical significance nor a trend toward improvement in the incidence of acute myocardial infarction, the need for ionotropic support, and ICU LOS, or hospital LOS with a random effects model meta-analysis of up to six trials. By consolidating the data from 10 trials, we observed a trend toward but not a significant reduction in LOS in ICU or hospital.

N-acetylcysteine is being used clinically for several decades. The main clinical uses for NAC to date include its mucolytic capacity in bronchi, as an antidote for acetaminophen toxicity, and as a protective agent against contrast-induced nephrotoxicity. NAC as a protective agent against reperfusion injury was first reported in 1992 by Sochman and Peregrin (6, 67, 68), who discovered total recovery of left ventricular function after acute myocardial infarction when NAC was administered along with the coronary artery thrombolysis during the PCI. Multiple RCTs have been published since to address possible beneficial effects of NAC during the coronary artery reperfusion. Twenty-eight of these RCTs reviewed in this study revealed a trend toward the improvement in several clinical measures, with a correlation to reduction of ROS or lipid peroxidation. The correlation approach provides evidence for the mechanistic basis of the observed benefit of NAC.

### Strengths and Limitations

We have included three types of coronary artery revascularization procedures for the clinical practice, PCI, thrombolytics, and CABG. This differs from the other published meta-analyses, which focused on one type of reperfusion procedure. Additionally, we have evaluated the most common clinical measures, cTn or CK-MB, LVEF, POAF, and ICU or hospital LOS, and provided a correlation for the levels of antioxidants or ROS to the clinical measures. This differentiates our study from the other published meta-analyses.

The included RCTs were from multiple countries, with most trials having a placebo control. There were minimal losses to follow-up across the trials. The data were generated from multiple healthcare centers with multi-ethnicities due to a diverse distribution of recruitment among the different countries. Additionally, none of the RCTs presented here were funded by a for-profit organization and the risk of bias was low in most of the trials.

The negative factors affecting our analysis power include limited regions of the trials, sample size, gender distribution, and substantial heterogeneity. While there was no restriction on the country or language for trial inclusion, over 50% of the evaluated studies originated from Turkey (10 trials) or Iran (5 trials), and none of the trials were carried out in the United States. Although many factors may explain the uneven distribution for the trial origins, the genetic background in association with a unique region, and the differences in socioeconomic status for the healthcare provision may prohibit extrapolation of the findings to all case scenarios worldwide. Additionally, most of the included trials had an enrollment below 100 individuals. The participants were mostly middle-aged men, prohibiting the generalization to other age groups or female patients.

We have detected a large between-study heterogeneity in most of the outcome measures, with I^2^ varying from 35 to 94% (Figure 3). Several variables in the trials contributed to the substantial heterogeneity: (a) non-uniform coronary reperfusion procedures, with either PCI, CABG, or thrombolysis in different trials; (b) the dosage and the route of NAC administration differed among the trials, with three trials using the low doses of NAC, 0.3–0.6 g (39, 43, 44); (c) the patient populations carried distinctive diagnoses, from acute coronary syndrome requiring an emergency reperfusion procedure to stable coronary artery diseases treated with an elective reperfusion protocol; (d) a lack of information on timing from the onset of chest pain to the reperfusion procedures. The large regional differences in such timing may affect the clinical outcome of reperfusion and NAC treatment; and (e) the healthcare facility and supportive infrastructure among the different countries or regions may influence the clinical outcome. If it had been possible to increase the sample sizes or reduce the heterogeneity, the statistical analyses would likely have yielded the *p*-values indicating significant differences supporting the benefit of NAC on multiple clinical outcome measures.

### Clinical Implications

Our findings suggest a trend toward the benefit of NAC treatment. The trend in the reduction of cTn suggests a potential reduction of cardiac injury by NAC. It is important to note that NAC, despite its low cost and multiple clinical implications already, is not free of side effects. Nausea and vomiting may be associated with an unpleasant odor during oral intake. For intravenous NAC, an anaphylactoid reaction occurs in 8.2% cases, such as cutaneous (acute flushing, pruritus, and rash) or systemic symptoms (bronchospasm, angioedema, hypotension, and chest pain) (69, 70). Additionally, NAC may have a negative impact on hemostasis in the patients under certain conditions. In a *post-hoc* analysis of an RCT of NAC in the patients undergoing cardiac surgery with an estimated glomerular filtration rate of ≤60 ml/min, administration of NAC (100 mg/kg IV bolus, followed by 20 mg/kg/h until 4 h after CABG) was associated with a greater blood loss and an increased need for transfusions (71). Therefore, the benefit of NAC remains to be fully established with larger controlled clinical trials measuring multiple clinical end-points. The risk vs. benefit analysis in such a trial would also be needed.

If well done, the RCTs with large numbers of patients were shown to be positive, then the addition of antioxidant therapy to the patients following reperfusion therapy or cardiopulmonary bypass would be a simple and inexpensive therapy. NAC, vitamin C, and other antioxidant agents are generic, inexpensive, generally safe, and would presumably be administered for a relatively short period of time, possibly hours to days. The long-term clinical implications of such therapy are not yet known and would need to be assessed.

## Data Availability Statement

The original contributions presented in the study are included in the article/Supplementary Material, further inquiries can be directed to the corresponding author.

## Author Contributions

SK: study design, building search strategy for six electronic databases, acquisition of data, qualitative analysis, interpretation of data, writing and drafting the manuscript, and coordinating the project. AC: independently reviewed the literature and evaluated all the selected trials, validated the acquisition of data, provided input, and edit to the manuscript. YL: quantitative analysis and conducted meta-analysis. LA: supervising statistician and manuscript editing. JA: a practicing cardiologist who helped with the clinical interpretation of the data, and manuscript editing. QC: initiated the conception, supervised the study, reviewed the literature, and revised the manuscript. All the authors edited and have approved this version of manuscript to be published.

## Funding

Research works under QC's direction are supported by NIH R01 GM125212, R01 GM126165, Holsclaw Endowment, and The University of Arizona College of Pharmacy start-up fund.

## Conflict of Interest

The authors declare that the research was conducted in the absence of any commercial or financial relationships that could be construed as a potential conflict of interest.

## Publisher's Note

All claims expressed in this article are solely those of the authors and do not necessarily represent those of their affiliated organizations, or those of the publisher, the editors and the reviewers. Any product that may be evaluated in this article, or claim that may be made by its manufacturer, is not guaranteed or endorsed by the publisher.
