# Supplementary material for: N-Acetylcysteine for Cardiac Protection During Coronary Artery Reperfusion: A Systematic Review and Meta-Analysis of Randomized Controlled Trials
Source: Front Cardiovasc Med. 2021 Nov 19;8:752939. doi: 10.3389/fcvm.2021.752939 (PMC8640098; doi:10.3389/fcvm.2021.752939)
Supplement: Supplementary file 1 [file Data_Sheet_1.pdf]

**Supplements**

Search Strategies for multiple databases

***PubMed (NCBI)***

Number of results: 89

| Search | Term | Results |
|---|---|---|
| 1 | Myocardial Reperfusions[MeSH Terms] | 529 |
| 2 | Myocardial Reperfusion[MeSH Terms] | 529 |
| 3 | T-Plasminogen Activator | 55097 |
| 4 | TTPA[MeSH Terms] | 2055 |
| 5 | Activase[MeSH Terms] | 2055 |
| 6 | Alteplase[MeSH Terms] | 2055 |
| 7 | Percutaneous Transluminal Coronary Angioplasty[MeSH Terms] | 2157 |
| 8 | Coronary Balloon Angioplasty[MeSH Terms] | 2157 |
| 9 | Coronary Balloon Angioplasties[MeSH Terms] | 2157 |
| 10 | Transluminal Coronary Balloon Dilation[MeSH Terms] | 2157 |
| 11 | Percutaneous Coronary Revascularization[MeSH Terms] | 4821 |
| 12 | Percutaneous Coronary Intervention[MeSH Terms] | 4821 |
| 13 | Coronary Artery Bypass Grafting[MeSH Terms] | 4046 |
| 14 | Aortocoronary Bypass[MeSH Terms] | 4046 |
| 15 | Coronary Artery Bypass Surgery[MeSH Terms] | 4046 |
| 16 | Coronary Artery Bypass[MeSH Terms] | 4046 |
| 17 | OR/1-16 | 10273 |
| 18 | Acetylcysteine[MeSH Terms] | 2287 |
| 19 | 17 AND 18 | 53 |

***Embase***

Number of results: 122

| Search | Terms | Results |
|---|---|---|
| 1 | 't-plasminogen activator':ab | 18 |
| 2 | 'ttpa':ab | 130 |
| 3 | 'activase':ab | 442 |
| 4 | 'alteplase':ab | 3309 |

| | | |
|---|---|---|
| 5 | 'percutaneous transluminal coronary angioplasty':ab | 6374 |
| 6 | 'coronary balloon angioplasty':ab | 302 |
| 7 | 'coronary balloon angioplasties':ab | 1 |
| 8 | 'transluminal coronary balloon dilation':ab | 0 |
| 9 | 'percutaneous coronary revascularization':ab | 487 |
| 10 | 'percutaneous coronary intervention':ab | 46799 |
| 11 | 'coronary artery bypass grafting':ab | 25471 |
| 12 | 'aortocoronary bypass':ab | 1856 |
| 13 | 'coronary artery bypass surgery':ab | 6914 |
| 14 | 'coronary artery bypass':ab | 47898 |
| 15 | OR/1-14 | 99246 |
| 16 | 'Acetylcysteine':ab | 14662 |
| 17 | 15 AND 16 | 122 |

***Web of Science (Clarivate Analytics)***

Number of results: 432
Indexes: SCI-EXPANDED, SSCI, A&HCI, CPCI-S, CPCI-SSH, BKCI-S, BKCI-SSH, ESCI, CCR-EXPANDED, IC.

| Search | Terms | Results |
|---|---|---|
| 1 | TS=t-plasminogen activator | 12 |
| 2 | TS= ttpa | 133 |
| 3 | TS=activase | 1054 |
| 4 | TS=alteplase | 4409 |
| 5 | TS=percutaneous transluminal coronary angioplasty | 6648 |
| 6 | TS=coronary balloon angioplasty | 12779 |
| 7 | TS=coronary balloon angioplasties | 12779 |
| 8 | TS=transluminal coronary balloon dilation | 124 |
| 9 | TS=percutaneous coronary revascularization | 13284 |
| 10 | TS=percutaneous coronary intervention | 51,343 |
| 11 | TS=coronary artery bypass grafting | 36549 |
| 12 | TS=aortocoronary bypass | 2335 |
| 13 | TS=coronary artery bypass surgery | 35943 |

| | | |
|---|---|---|
| 14 | TS=coronary artery bypass | 50131 |
| 15 | OR/1-14 | 112568 |
| 16 | TS=Acetylcysteine | 18025 |
| 17 | 15 AND 16 | 432 |

***CINAHL (EBSCOhost)***

Number of Results: 24

Limiter: Human

Expanders: Apply equivalent subjects

Search modes: Boolean/Phrase

| Search | Term | Results |
|---|---|---|
| 1 | AB t-plasminogen activator | 1 |
| 2 | AB ttpa | 5 |
| 3 | AB activase | 6 |
| 4 | AB alteplase | 376 |
| 5 | AB percutaneous transluminal coronary angioplasty | 677 |
| 6 | AB coronary balloon angioplasty | 127 |
| 7 | AB coronary balloon angioplasties | 127 |
| 8 | AB transluminal coronary balloon dilation | 0 |
| 9 | AB percutaneous coronary revascularization | 279 |
| 10 | AB percutaneous coronary intervention | 4740 |
| 11 | AB coronary artery bypass grafting | 2228 |
| 12 | AB aortocoronary bypass | 57 |
| 13 | AB coronary artery bypass surgery | 2470 |
| 14 | AB coronary artery bypass | 5223 |
| 15 | OR/1-14 | 10113 |
| 16 | TX Acetylcysteine | 992 |
| 17 | 15 AND 16 | 24 |

***ClinicalTrials.gov***

Number of Results: 10

Applied filters: Interventional Studies (Clinical Trials), Adults (18-64

Study Results: All Studies

| Search | Term | Results |
|---|---|---|

| | | |
|---|---|---|
| 1 | Condition or disease: Myocardial infarction | 2432 |
| 2 | Condition or disease: STEMI | 674 |
| 3 | Condition or disease: NSTEMI | 208 |
| 4 | Condition or disease: Coronary artery disease | 7663 |
| 5 | Condition or disease: Ischemic Heart Disease | 7633 |
| 6 | Condition or disease: Angina Pectoris | 816 |
| 7 | Condition or disease: Stable Angina | 287 |
| 8 | Condition or disease: Unstable Angina | 223 |
| 9 | Condition or disease: OR/ 1-9 | 7921 |
| 10 | Intervention/treatment: Acetylcysteine | 424 |
| 11 | 9 AND 10 | 10 |

***Cochrane Library (Wiley)***

Number of results: 81

Search limits: Trials

| No. | Query | Results |
|---|---|---|
| 1 | "t-plasminogen activator":ab | 4 |
| 2 | "ttpa":ab | 17 |
| 3 | "activase":ab | 20 |
| 4 | "alteplase":ab | 758 |
| 5 | "percutaneous transluminal coronary angioplasty":ab | 775 |
| 6 | "coronary balloon angioplasty":ab | 37 |
| 7 | "coronary balloon angioplasties":ab | 37 |
| 8 | "transluminal coronary balloon dilation":ab | 0 |
| 9 | "percutaneous coronary revascularization":ab | 77 |
| 10 | "percutaneous coronary intervention":ab | 7633 |
| 11 | "coronary artery bypass grafting":ab | 6470 |
| 12 | "aortocoronary bypass":ab | 315 |
| 13 | "coronary artery bypass surgery":ab | 1299 |
| 14 | "coronary artery bypass":ab | 7893 |
| 15 | OR/1-14 | 16276 |
| 16 | "Acetylcysteine":ab | 1456 |
| 17 | 15 AND 16 | 81 |

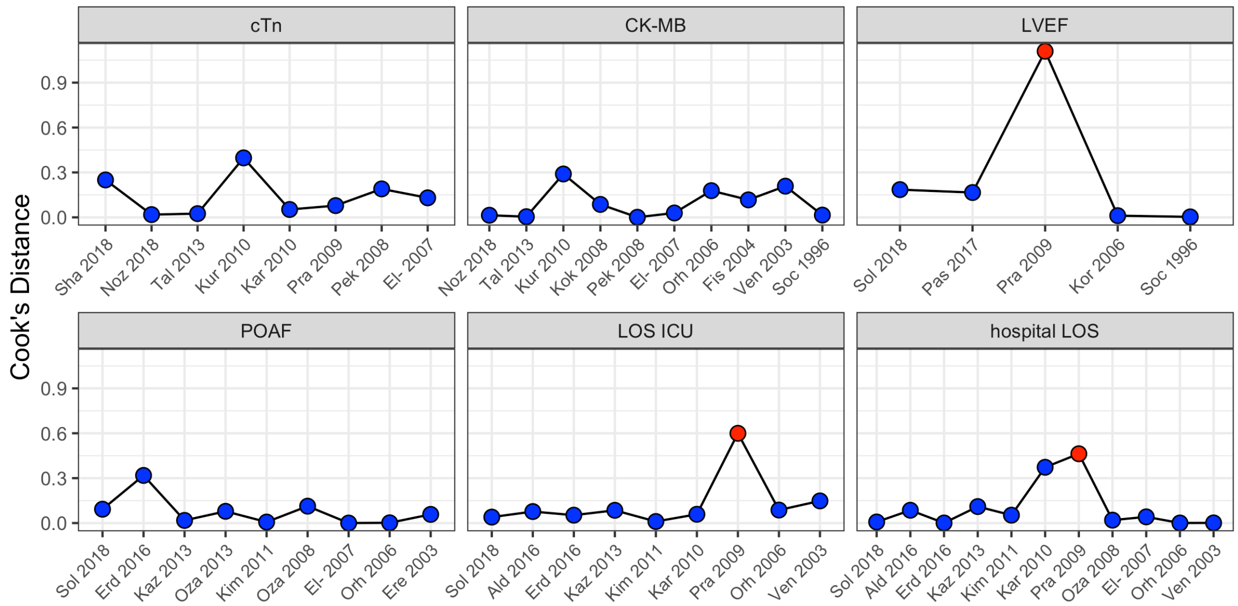


**Figure S1. Diagnostic plots for a potential influential trial.** X-axis represents the studies used in each measure for meta-analyses. Y-axis is the Cook's distance for each trial. If the Cook's distance is greater than 0.45, the corresponding trial is highlighted in red, as an indicator of a potentially influential trial.

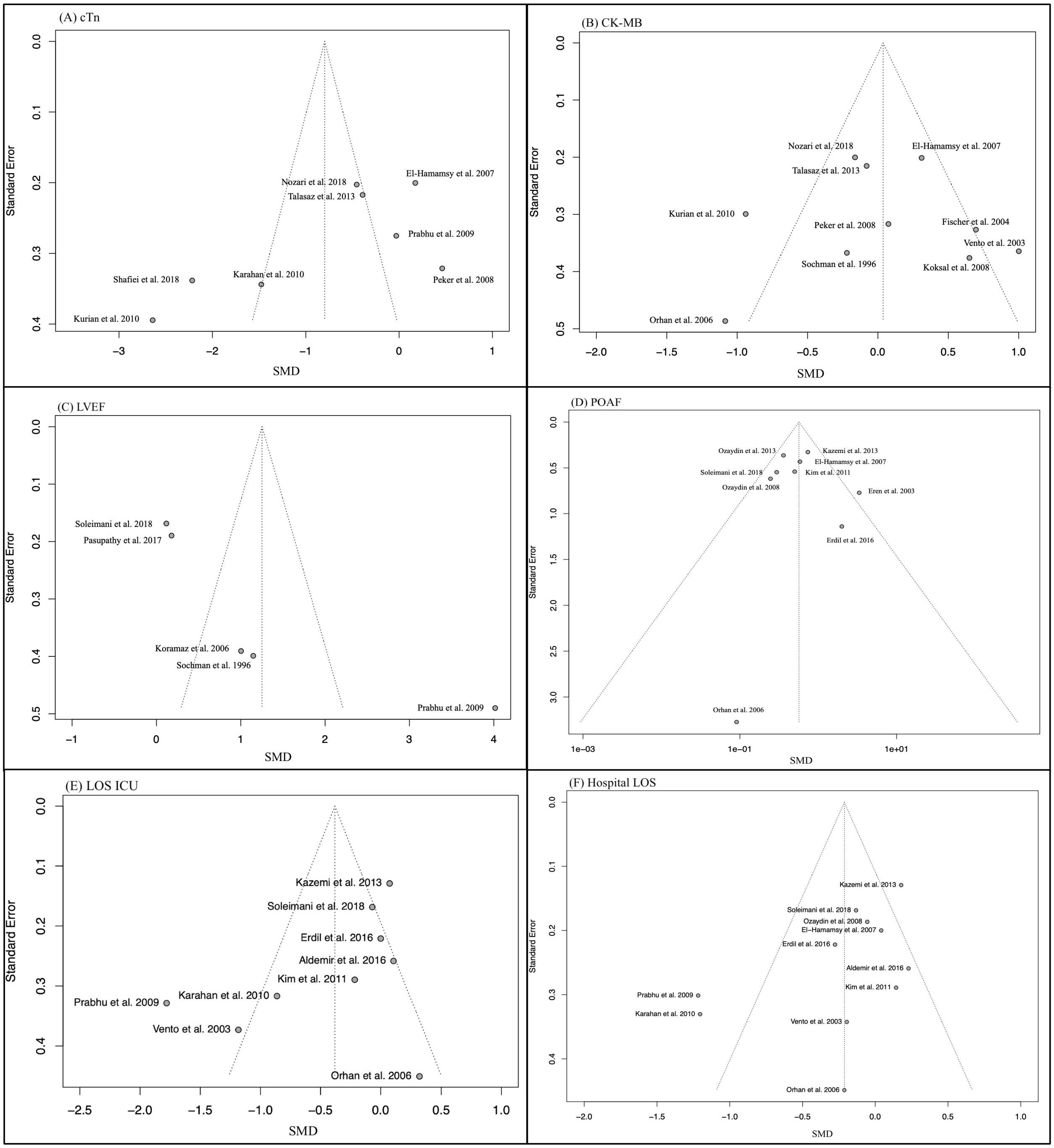


**Figure S2. Funnel plot for (A) cTn, (B) CK-MB, (C) LVEF, (D) POAF, (E) LOS ICU, (F) Hospital LOS**. Each dot represents a trial. The x-axis represents the SMD for each study, whereas the y-axis indicates the standard error for each trial. The middle dashed line shows the pooled SMD, with two other dashed lines indicate 95% CI for the pooled SMD. When there is no publication bias, the assembly of trials should form a roughly symmetrical, upside-down funnel.

**Table S1. Comparison of Meta-Analysis Results for Inclusion versus Exclusion of the Potential Influential Trial.**

| Outcome | Analysis | Pooled effect size | 95% CI | p value | Heterogeneity |
|---|---|---|---|---|---|
| LVEF | Include all studies | 1.251 | [-0.701, 3.204] | 0.150 | 94% |
| | Remove Prabhu et al. 2009 | 0.515 | [-0.324, 1.353] | 0.146 | 68% |
| LOS ICU | Include all studies | -0.381 | [-0.913, 0.151] | 0.137 | 81% |
| | Remove Prabhu et al. 2009 | -0.200 | [-0.601, 0.200] | 0.276 | 61% |
| Hospital LOS | Include all studies | -0.213 | [-0.541, 0.116] | 0.180 | 70% |
| | Remove Prabhu et al. 2009 | -0.109 | [-0.380, 0.162] | 0.386 | 52% |

Prabhu, et al., 2019 trial was detected as a potential influential trial for LVEF, LOS ICU and hospital LOS but not for cTn, CK-MB or POAF (Fig 4). LVEF: left ventricular ejection, LOS: length of stay.

**Table S2. Test Results for Asymmetry in the Funnel Plots.**

| Outcome | t statistics | p value |
|---|---|---|
| cTn | -2.155 | 0.075 |
| CK-MB | 0.001 | 0.999 |
| LVEF | 2.987 | 0.058 |
| POAF | 0.530 | 0.614 |
| LOS ICU | -1.841 | 0.108 |
| Hospital LOS | -1.968 | 0.081 |

The Egger's regression test was employed for evaluating the asymmetry of continuous clinical outcome measures, cTn, CK-MB, LVEF, LOS ICU, and hospital LOS, whereas the Peters' regression test was used for the binary outcome measure POAF. A p value greater than 0.05 indicates the asymmetry of the funnel plot is not significant at 5% level, and there is no publication bias related to the trials with a small sample size. cTn: cardiac troponins, LVEF: left ventricular ejection, POAF: post-operative atrial fibrillation, LOS: length of stay.
